# Small-Molecule Targets in Tumor Immunotherapy

**DOI:** 10.1007/s13659-018-0177-7

**Published:** 2018-07-05

**Authors:** Hui-Fang Zhu, Yan Li

**Affiliations:** 1 State Key Laboratory of Phytochemistry and Plant Resources in West China, Kunming Institute of Botany, Chinese Academy of Sciences, 132# Lanhei Road, Kunming, 650201 Yunnan People’s Republic of China; 2 University of Chinese Academy of Sciences, Beijing, 100049 People’s Republic of China

**Keywords:** Cancer immunotherapy, IDO1, PD(L)-1, NKG2DL, STING, TLRs

## Abstract

Cancer immunotherapy has been widely recognized as a powerful approach to fight cancers. To date, over 50 phase III trials in cancer immunotherapy are in progress. Among the many immunotherapy approaches, immune checkpoint therapy has attracted considerable attention. The reported clinical success of targeting the T cell immune checkpoint receptors PD-1 or CTLA4 by antibodies blockade in advanced stages of cancers has demonstrated the importance of immune modulation. But antibodies-based immunotherapy confronted with some disadvantages, such as immunogenicity, stability, membrane permeability, and production cost. Therefore, alternative approaches including small-molecule-regulated immune response are being introduced. In this review, we focused on some of the key intracellular pathways where small-molecule therapeutic is potential and attractive, which highlights the great potential of natural products in this field.

## Introduction

After decades of research, tumor immunotherapy has convincingly demonstrated to be a feasible approach to treat various cancers. And the most remarkable responses in clinical experience have been seen with blockade of checkpoint proteins in melanoma and non-small-cell lung cancer (NSCLC). These impressive results have already led to the regulatory approval of several monoclonal antibodies targeting the cell-surface proteins, such as nivolumab and pembrolizumab as PD-1 inhibitors, ipilimumab as CTLA4 inhibitor and durvalumab as PD-L1 inhibitor [1–4]. Nonetheless, antibody drugs are not all for cancer immunotherapy, conspicuously missing from this class of therapies are traditional small-molecule drugs, which have previously served as the backbone of targeted cancer therapies.

Modulating the immune system through small molecules offers several unique advantages as follows: (1) oral bioavailability; (2) penetration of physiological barriers and easier exposure within the tumor microenvironment; (3) diverse and well-understood formulation and dosing options to alleviate pharmacokinetic and/or pharmacodynamics challenges and enabling titration of drug exposure. Another compelling advantage of small-molecule drugs is the lower in cost. Thus, the development of small-molecule drugs are complementary and potentially synergistic with large biological molecules, and is now coming into spotlights in the field of immunotherapy.

Here, we described the rationale for using small-molecule drugs targeting some of the key intracellular proteins or activities as immuno-therapies in cancer, including amino acid catabolism, T cell immune checkpoint proteins, NK cell sensitization effect and pattern recognition receptors. We offer considerations regarding the future promise of their utility both as single agents and in combination with other cancer medicines.

## IDO Inhibitors

Indoleamine-2,3-dioxygenase 1 (IDO1) is a monomeric 45 kDa hemoglobin-containing oxidase, it is one key dioxygenase in the process of tryptophan-kynurenine metabolism [5], and usually acts as one immune escape strategy for cancer cells [6, 7]. Recent investigations have shown that IDO1 upregulation is associated with increased Treg numbers in malignant melanoma [8]. It also has been identified that Treg can participate in the silencing of T cells response by acting to enforce a dominant negative regulation on T cells activation, proliferation and cytokine production [9]. By coopting IDO1 activity, tumor cells can effectively mask their rapid cellular growth and evade the host immune surveillance. Therefore, inhibition of IDO1 activity by small-molecule drug was one valuable strategy for cancer patient to re-establish immunogenic response.

Since the participation of IDO1 in oncogenesis was first uncovered in 2003, thousands of bioactive small molecule inhibitors have been reported, nevertheless, to date, only five compounds are undergoing clinical trials (Table 1). Among these, 1-Methyl-d-tryptophan (D1MT) is in phase I/II clinical studies at NewLink Genetics Cop. for the treatment of metastatic prostate cancer, acute myeloid leukemia, primary malignant brain tumors, metastatic pancreatic cancer, metastatic breast cancer, metastatic melanoma, as well as NSCLC. The *N*-hydroxyamidine INCB024360 is in phase II/III clinical trials at Incyte Corp., used as a monotherapy as well as in combination with various antibodies, for the treatment of advanced or metastatic cancers. Another IDO1 inhibitor, the imidazole GDC-0901 (navoximod) is in phase I clinical trials at NewLink Genetics Corp. in collaboration with Genentech Inc. in subjects with recurrent advanced solid tumors. The in vivo study revealed that treatment with GDC-0901 led to a significant reduction of tumor size which was highly relevant to its functional immune response. Recently, two 2nd/3rd generation IDO1 inhibitors, PF-0684003 (EOS-200271) and BMS-986205 (ONO-7701), have also entered into clinical trials within the last few months. PF-0684003, from Pfizer Inc./iTeos Therapeutics SA, is in phase I clinical trials for the treatment of patients with grade IV glioblastoma or grade III anaplastic glioma. BMS-986205 is being evaluated at Bristol-Myers Squibb Co. in phase I/II advanced cancer.

**Table 1:**
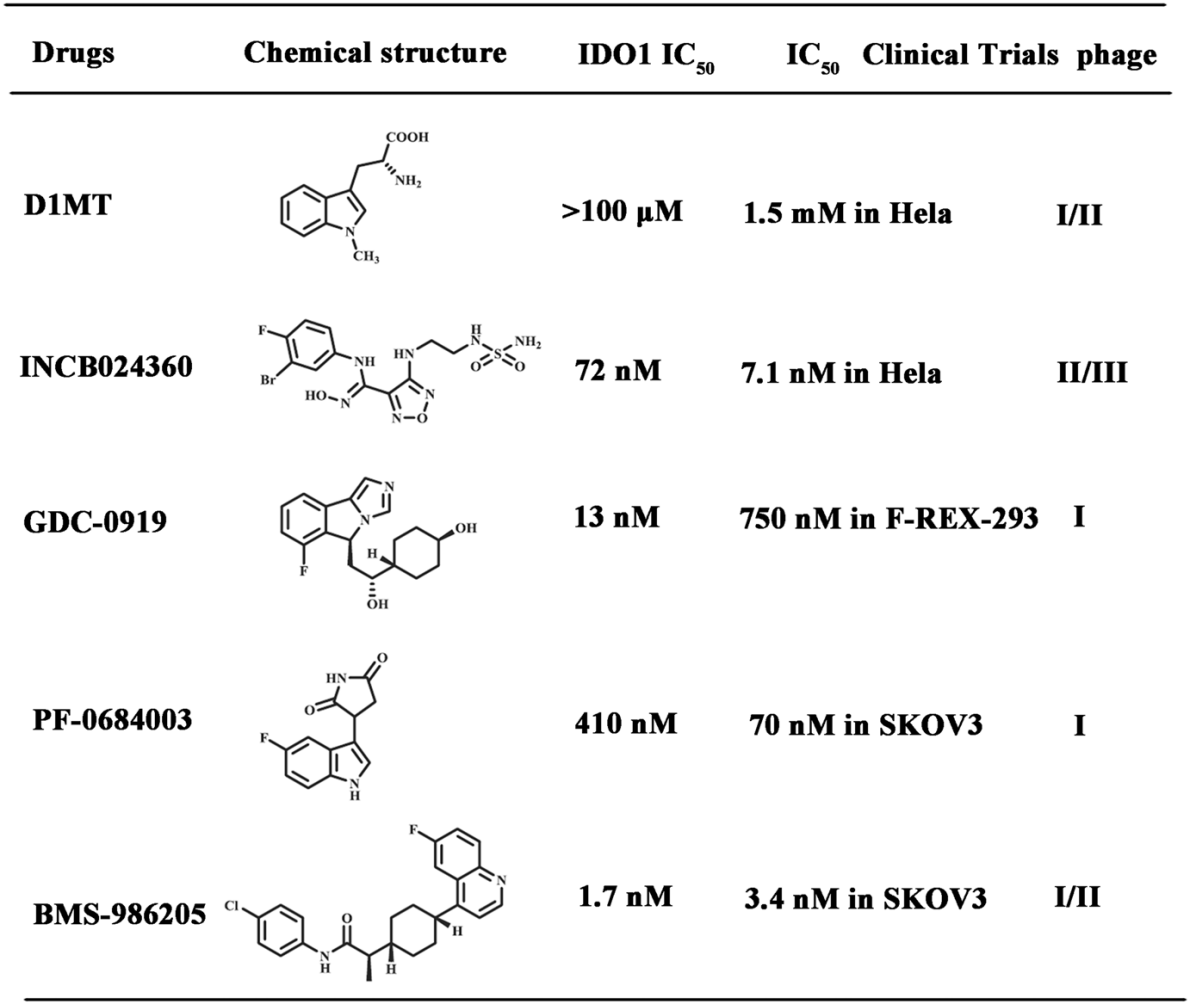
IDO1 inhibitors ongoing clinical trials

In an effort to obtain more clinical drugs targeting IDO1, more comprehensive understanding of the active site of IDO1 and the structures of existing potent IDO1 inhibitors are necessary.

## PD-1/L1 Inhibitors

Programmed cell death 1 ligand (PDL-1) is expressed on tumor cell surface and acts as immune checkpoint protein, engaging its receptor PD-1 on activated T cells will cause T cell exhaustion. Therefore, checkpoint blockade is potential for sustained tumor protection.

Antibody-mediated immune checkpoint blockade has shown great clinical benefit in several tumor types [10]. Anti-PD-1/PDL-1 therapy in late-stage metastatic NSCLC and head and neck squamous cell carcinoma have supported as standard of care of these regimens [11–13]. Nonetheless, responsiveness is generally limited to only 20–40% of patients and often most individuals fail therapy [14]. Given the lack of success with empirical combination therapy, as well as production cost, stability and immunogenicity, attention has turned to develop more rational and individualized approaches to boost clinical response rates.

Small-molecule PD-1/PD-L1 inhibitors have only begun to be identified. Until recently, few small-molecule PD-1/PD-L1 inhibitors have been reported. To our knowledge, only one patented PD-1/PD-L1 drug-like inhibitor CA-170 has undergone clinical trials (Table 2). CA-170 was screened from B7 immunoglobulin superfamily members in a checkpoint protein interaction surface simulant library, which could selectively target PD-L1 and VISTA. In vitro studies, CA-170 exhibited cross-species antagonistic effects on PD-1/PDL-1 signaling pathways, which showed similar anti-tumor effects to those of antibodies-based PD(L)-1 blockade drugs. Its oral administration promoted tumor infiltration and peripheral T cell viability in a dosage-dependent manner with an EC_50_ of 17 nM in advanced solid tumors. In preclinical toxicology studies, CA-170 represented oral safety on a once daily dosing schedule in lymphomas. While the structure of CA-170 has not yet been disclosed.

**Table 2:** PD(L)-1 inhibitors ongoing clinical trials

Drug	Indications	Ec_50_	Clinical trials phage
CA-170	Advanced in solid tumors and lymphomas	17 nM	1

Checkpoint regulators generally work in protein–protein interaction (PPI) manner, and the preferences to block PPI are monoclonal antibodies than small-molecules. Due to the typical flatness, large size, and non-contiguity of the interface between the interacting proteins and the flexibility of their surfaces, identification of small-molecule inhibitors disrupting PPI is a challenge. Success of small molecule inhibitor of PD1/PD-L1 indicates PPI is targetable.

## NKG2D Ligands Inducer

NK (natural killer) cells play a crucial role in the surveillance of malignant cells, whose activity are not required the prior sensitization of special antigens, but dependent on the regulatory balance of inhibitory and activating receptors [15]. Among these, the natural-killer group 2 member D (NKG2D) receptor and its ligands is one of the most characterized interaction pair [16]. And the engagement of ligands on tumor cells represent a promising therapeutic strategy against cancer [17, 18]. To date, most of NKG2D ligands inducers are concentrated upon histone deacetylase (HDAC) inhibitor, such as sodium valproate (VPA), trichostatin A (TSA) and entinostat [19–21]. In addition, some chemotherapy agents like cisplatin or proteasome inhibitor MG132 were also reported to be able to induce the expression of NKG2D ligands [22, 23]. Besides, a natural product matrine was also demonstrated to up-regulate the expression of NKG2D ligands [24, 25]. But to date, none of the NKG2D ligands inducers are under clinical tests. More basic research upon NK cell and lead compounds are needed to promote this process.

## STING Agonist

Innate immune responses play an important role against cancer during the process of tumorigenesis. And through a series of knockout mice and other ancillary studies, researchers found that the STING pathway is the main bridge to this spontaneous antitumor immunity [26, 27]. STING is an ER-associated trans-membrane protein, acting as a novel nucleic acid sensor, which is activated not only by pathogen-derived nucleic acids, but also self-DNA released by host apoptotic or necrotic cells detected by dendritic cells [26, 28, 29]. After the activation of STING signaling, the production of type I IFNs will lead to a powerful immune cascade like inflammatory cytokines and other elements [26, 30].

The use of STING agonist as a cancer treatment is disclosed in the patent US20160287623 (Pub. Date October 6, 2016). Natural STING ligands are cyclic dinucleotides [31]. Currently, at least two STING agonists, ADU-S100/MIW815 and MK1454 are in early-stage clinical trials [32, 33]. ADU-S100 is a synthetic cyclic dinucleotide STING agonist, which is an effective stimulator for the induction of IFN-β in vitro in human peripheral blood mononuclear cells (PBMCs). The clinical trials for MK-1454 are ongoing by alone or in combination with pembrolizumab in participants with advanced/metastatic solid tumors or lymphomas. To date, no detail results were posted.

## TLRs Agonist

Toll-like receptors (TLRs) are type I transmembrane proteins that function as microbial pattern recognition molecules, which are expressed on antigen-presenting cells. The activation of TLRs stimulated by pathogen-associated molecular patterns leads to a rapid innate immune response and also induces appropriate adaptive immune responses. The family of TLRs may be considered the seminal immuno-oncology targets if Coley’s toxins are supposed to be the original immunotherapy for cancer [34].

Generally, endogenous ligands for TLRs are derived from viruses or bacteria, as well as a variety of non-classical molecular patterns including double-stranded RNA and polynosinic:polycytidylic acid (poly I:C) (TLR3), lipopolysaccharide (TLR4), and un-methylated CpG oligodeoxynucleotides (TLR9). Clinical trials of TLR agonists for cancer indications have examined agonists of endosomal TLRs (TLR3, TLR7, TLR8 and TLR9) and utilized as either vaccine adjuvants or monotherapy. The anti-tumor effects of TLR7 and TLR8 agonists are primarily by mediating the activation of dendritic cells and natural killer cells, and suppressing activation of Treg cells. Small-molecule heterocyclics, such as imidazoquinolines are recognized as nucleoside agonists by TLR7 and TLR8 [35]. And imiquimod is the first approved LR7 and TLR8 agonist by the FDA in 1997 for the treatment of genital warts, and for the topical treatment of basal cell carcinoma in 2004 [36]. Several synthetic oligodeoxynucleotides agonists for TLR9 such as IMO-2055, CPG 7909 and MGN1703, are currently in development for the treatment of colorectal cancer, NSCLCs and renal cell carcinoma [37].

Once engaged, TLRs will trigger a strong pro-inflammatory cytokine response following by a stimulation of immune surveillance, which could be important for the elimination of cancer. But until recently, due to high polarity, presence of charged species or poor hydrolytic stability for TLRs ligands, there are no any clinically evaluated oral small-molecule TLRs agonists. Therefore, more research about TLRs agonists are needed for exploitation of orally bioavailable drug-like analogs.

## Future Directions

In this review, we have highlighted some small-molecule drugs and targets that have the potential to stimulate an effective immune response to cancer. As monotherapy, these drugs are not universally effective across tumor types or in all patients, therefore, exploring of more potential small-molecule drugs and establishing combination agent strategies is the general trend upon cancer immunotherapies in the future.

Natural products are one important part of potential drugs to treat cancer, which usually have multiple targets and few adverse reactions and less drug resistance. Therefore, the antitumor activities of natural products have attracted extensive research, and many natural products act as leading compounds in the exploitation of antitumor drugs. But the application of natural products on immunomodulation are few. Thereby, performing more laboratory and clinical studies upon immunomodulatory activity is important for future natural product study.
